# Neural Activation in Humans during a Simple Motor Task Differs between BDNF Polymorphisms

**DOI:** 10.1371/journal.pone.0096722

**Published:** 2014-05-14

**Authors:** Lizbeth Cárdenas-Morales, Georg Grön, Eun-Jin Sim, Julia C. Stingl, Thomas Kammer

**Affiliations:** 1 Department of Psychiatry, University of Ulm, Ulm, Germany; 2 Max Planck Institute for Neurological Research, Cologne, Germany; 3 Neurological Department, University of Cologne, Cologne, Germany; 4 Institute of Pharmacology of Natural Products and Clinical Pharmacology, University of Ulm, Ulm, Germany; 5 Research Department, Federal Institute for Drugs and Medical Devices, Bonn, Germany; University Pablo de Olavide, Spain

## Abstract

The BDNF Val66Met polymorphism has been linked to decreased synaptic plasticity involved in motor learning tasks. We investigated whether individual differences in this polymorphism may promote differences in neural activity during a two-alternative forced-choice motor performance. In two separate sessions, the BOLD signal from 22 right-handed healthy men was measured during button presses with the left and right index finger upon visual presentation of an arrow. 11 men were Val66Val carriers (ValVal group), the other 11 men carried either the Val66Met or the Met66Met polymorphism (Non-ValVal group). Reaction times, resting and active motor thresholds did not differ between ValVal and Non-ValVal groups. Compared to the ValVal group the Non-ValVal group showed significantly higher BOLD signals in the right SMA and motor cingulate cortex during motor performance. This difference was highly consistent for both hands and across all four sessions. Our finding suggests that this BDNF polymorphism may not only influence complex performance during motor learning but is already associated with activation differences during rather simple motor tasks. The higher BOLD signal observed in Non-ValVal subjects suggests the presence of cumulative effects of the polymorphism on the motor system, and may reflect compensatory functional activation mediating equal behavioral performance between groups.

## Introduction

Brain derived neurotrophic factor (BDNF) is a neurotrophin involved in synaptic long-term plasticity mechanisms, neurogenesis, survival of motoneurons and neuronal cell migration [1], [2]. It is secreted in the pro-form (proBDNF), and subsequently converted to mature BDNF (mBDNF) by extracellular proteases. BDNF has a very high affinity to bind to the B-type of tropomyosin-kinase receptors (TrkB) which is thought to promote synaptic efficacy [3].

Val66Met is a single nucleotide polymorphism (SNP) in the human BDNF gene located on chromosome 11. In approximately 30% of the population [4], [5] this SNP is present in one or both alleles at codon 66 resulting in a variation between valine (Val) and methionine (Met). Val66Met SNP has been shown to affect intracellular trafficking of pro-BDNF and to alter the regulated release of activity-dependent BDNF within a period of at least 6 min [6], [7]. In humans this polymorphism has been associated with memory impairments, reduction in cortical volumes and neuropsychiatric disorders [4], [8], [9].

More recently, the Val66Met SNP has been associated with changes of human motor cortex excitability after training, short-term plasticity-like processes induced by non-invasive brain stimulation, and greater error rates during motor learning [10]–[12]. Differences in the organization of primary sensory and motor brain areas (S1/M1) after 25 min of short-term motor finger practice and after driving-based motor learning were reported in healthy carriers of the Met allele [12]. ValVal carriers showed a significantly increased spatial extent of neural activity in bilateral S1/M1 cortex during short-term motor learning and committed fewer mistakes when compared against Val/Met and Met/Met carriers. Interestingly, percent BOLD-signal change did not covary with this polymorphism [12].

By contrast, fMRI data obtained during virtual navigation memory tasks demonstrated no differences in performance between genotype groups, but showed variations in the height of activation of task-related brain regions [13]. In agreement with previous studies [4], [8] the Met allele was associated with a greater probability of involving the caudate nucleus to solve the task rather than the hippocampus [13]. These findings support the assumption of compensational neural recruiting to be present in Met carriers.

In the present study we made use of these previous results, however hypothesizing, that differences in neural activities between two different groups of SNP carriers could possibly exist already at a very basic level of rather simple motor performance. In a sample of 22 healthy participants repeated measurements of neural activity upon a two-alternative forced choice reaction task were performed to compare brain activity in BDNF ValVal and Non-ValVal (Val/Met and Met/Met) carriers using functional magnetic resonance imaging (fMRI). Importantly, to obtain an estimate of the stability of putative between-group differences fMRI was repeatedly performed at two separate days with a fixed interval of two days in-between. Cortical excitability was additionally measured using single pulse TMS.

## Methods

### Subjects

22 healthy, male volunteers aged between 19 and 33 years (mean age: 26.54±3.26 years) were recruited from a pool of subjects genotyped for the BDNF Val66Met SNP. While all Non-ValVal carriers identified were invited to participate, from the ValVal group a sample of equal size was randomly drawn. All invited participants took part in the study. They were right handed (0.87±0.13, mean score ± SD) according to the Edinburgh inventory [14]. Both subgroups of ValVal and Non-ValVal carriers with 11 subjects each did not differ on age (ValVal: 27.1±0.7 years; Non-ValVal: 26.1±1.1 years, *t*_(20)_ = 0.71, *p* = 0.48) and handedness (ValVal: 0.85±0.1; Non-ValVal: 0.90±0.1; *t*_(20)_ = −0.87, *p* = 0.4). MRI operators were blinded to the genotype. We restricted the study to male subjects in order to avoid signal fluctuation due to the influence of ovarian hormones (cf. [15], [16]). Exclusion criteria were metallic implants, a history of brain injury, the presence of major medical illness, neurological or psychiatric clinical history, intake of medication during the study, and history of drug intake. All participants gave their written informed consent for the experiments and were paid for participation. The project followed the Declaration of Helsinki and was approved by the Ethics Committee of the University of Ulm.

### Genotyping

Each subject's blood sample was genotyped for the BDNF Val66Met polymorphism using the following forward and reverse primers: 5′-actctggagagaatgg-3′; 5′-actactgagccctgga-3′ (Biomers, Ulm, Germany). Genomic DNA was extracted using QUIamp DNA Mini Kit (Quiagen, Hilden, Germany). Amplification reaction was performed in a total volume of 42 µl, containing 2 µl of genomic template, 10 µM of each primer, 2 mM deoxyribonucleotide triphosphate (dNTP), 2 µl dimethyl sulfoxide, 1,2 µl MgCl_2_, 10 x buffer and 1 U of Taq polymerase (Invitrogen, Carlsbad CA, USA). PCR conditions were incubation for 2 min at 94°C, followed by 35 cycles (40 sec at 94°C, 40 sec at 58°C and 2 min at 72°C) and 3 min at 72°C. PCR product was checked on a 2.5% agarose gel stained with ethidium bromide. Digestion of the PCR product was performed with the enzyme Eco 721 (Fermentas, Thermo Fischer Sci., Waltham, MA, USA). The reaction consisted of 1 µl enzyme and 2 µl buffer in 10 µl together with 10 µl of PCR product. In the final electrophoresis, the presence of the G allele produced two products, 72 bp and 99 bp, whereas the A allele produced 171 bp only.

### Motor cortical excitability measurements

In order to test for differences in motor cortical excitability we obtained individual resting and active motor thresholds (RMT and AMT, respectively) for the right abductor pollicis brevis (APB) muscle, applying single biphasic pulses over the left motor hot spot (Magpro X100 stimulator, MagVenture, Skovlunde, Denmark, figure-of-eight coil MC-B70). The coil was placed over the hot spot of left motor cortex with the handle pointing backward and laterally at a 45° angle to the sagittal plane. First upstroke of the induced biphasic current in the brain was antero-posterior. Resting motor threshold (RMT) was defined as the lowest stimulus intensity that elicited at least six responses ≥50 µV within 10 consecutive single pulses (cf. [17]). Active motor threshold (AMT) was defined as the lowest stimulus intensity that elicited a response ≥200 µV averaged from 10 consecutive single pulses during voluntary contraction (10% of maximum force, online measured and visualized as average of a quadratic mean amplitude, based on [18]).

### Magnetic resonance imaging

A 3-Tesla MRI head-only system (Magnetom Allegra, Siemens, Erlangen, Germany) was used for the experiment. For structural imaging high resolution T1-weighted anatomical images were obtained using a 3D MPRAGE sequence with repetition time (TR) 2.08 s, inversion time 1 s, echo time (TE) 3.93 ms, bandwidth (BW) 130 Hz/Pixel, flip angle 12°, matrix 256×256 pixels (1×1 mm^2^). The volume consisted of 256 contiguous slices of 1 mm thickness acquired in sagittal direction. Total scan time was about 7.5 min. For BOLD imaging T2*-weighted functional MR images were obtained using gradient echo echo-planar imaging in axial orientation along the AC-PC-line with TR = 2 s, TE = 36 ms, BW 3906 Hz/Pixel, flip angle 90°. In-planar matrix size was 64×64 pixels (3.6×3.6 mm^2^). The volumes consisted of 30 slices of 3 mm with a gap of 0.6 mm, resulting in isotropic voxel sizes. A total of 180 volumes were acquired for each scan lasting 6 min. The first two volumes of each scan were discarded to allow for T1 equilibration.

### Experimental setup

24 h prior to the first fMRI session RMT and AMT were obtained. The study design comprised two sessions performed on two different days separated by 48 h. On each testing day two fMRI sessions were performed starting at the same day time. The first fMRI session on each day started with an anatomical T1 scan in order to equilibrate the cardio-vascular system to the supine position and to get participants acquainted. A slow event-related design was applied to measure the BOLD fMRI signal during a two-alternative forced choice task. On average, every 25 s (±3 s jitter) an arrow appeared on the screen directing either to the left or to the right (MR-compatible LCD goggles, VisuaStim Digital Interface Box, Resonance Technology Inc. CA, USA). Upon appearance of this stimulus subjects had to press a button with the corresponding index finger of the left and right hand as fast as possible (Task written in Presentation, V 11.0 Neurobehavioral Systems Inc., San Francisco, CA, USA). As baseline condition subjects were asked to fixate on a white cross in the center of the screen. During each fMRI session of six minutes duration a total of 18 arrows (nine pointing left, nine pointing right) were presented in pseudo-randomized order. The duration of each session had been time-limited to 6 minutes since the minimum time required for activity-dependent BDNF secretion is 4 min [4]. Between the two fMRI sessions of each day subjects remained at rest and were asked to close their eyes for the duration of four minutes.

### Data analysis

#### Behavioral data

For the eight combinations of Hand by Day by Session median individual response times were averaged across groups and statistically compared using two sample t-tests.

#### Imaging Data

Image preprocessing and statistical analyses were performed using Statistical Parametric Mapping (SPM5, Wellcome Department of Cognitive Neurology, London, UK; http://www.fil.ion.ucl.ac.uk). First, data from each experimental session were temporally slice-time corrected and resliced. Second, functional images for each series were realigned in order to correct for small movements during each session. Third, functional images acquired during the fMRI sessions of one experimental day were spatially aligned and coregistered to the individual T1 image. Forth, all images were spatially normalized to a canonical T1 template in MNI (Montreal Neurological Institute) space. Finally, data were spatially smoothed with a 12 mm^3^ full width at half maximum isotropic Gaussian kernel.

Session separated regressors in the General Linear Model described the occurrences of the different motor responses of the right or left hand index finger in form of delta functions at each onset of a button press which were convolved with the canonical hemodynamical response function. Image time series were scaled to a grand mean of 100 over all voxels and volumes. Low frequency drifts were removed by a high pass filter with a cut-off of 128 s. Parameter estimation was corrected for serial correlations by use of a first-order autoregressive model.

In individual first-level analyses for each of the four different sessions the difference of neural activity upon right and left hand motor responses against baseline was estimated using a one-tailed *t*-contrast. These eight individual contrast images were transferred to a group analysis. An analysis of variance was set up with three factors: *group* (ValVal, Non-ValVal), *repetition* (4 levels), and *hand* (left, right). Group differences were tested by means of an appropriate two-tailed *F*-test contrasting neural activity upon both motor responses per each of the four sessions.

## Results

### Motor threshold and behavioral responses – choice reaction time task

No significant difference was observed neither for RMT (ValVal: 37.5±2.6%; Non-ValVal: 36.2±1.9%; means ± SEM, *t*_(20)_ = 0.42, *p* = 0.68) nor for AMT (ValVal: 26.9±1.7%; Non-ValVal: 26.3±1.8%; *t*_(20)_ = 0.26, *p* = 0.8).

Mean reaction times obtained from the fMRI task were compared for each hand separately among scans (Table 1). Statistical analysis revealed no significant difference between groups by means of Student's *t*-test (Bonferroni-corrected, *see* Table 1).

**Table 1:** Mean reaction times per hand and session.

Hand	Day	Session	ValVal RT	Non-ValVal RT	*t*-value	*p*
left	1	1	418±52	426±65	−0.33	1
left	1	2	447±60	418±49	1.22	1
left	2	1	410±41	442±59	−1.50	1
left	2	2	431±43	464±35	−1.94	0.53
right	1	1	436±58	444±49	−0.33	1
right	1	2	439±46	416±31	1.38	1
right	2	1	407±53	436±70	−1.09	1
right	2	2	434±36	450±70	−0.67	1

Mean values ± SD in milliseconds. *t*-values and *p* from Students *t*-test comparing ValVal and Non-ValVal. *p* is corrected according to Bonferroni. RT: reaction times.

### Functional imaging – choice reaction time task

**Figure 1:**
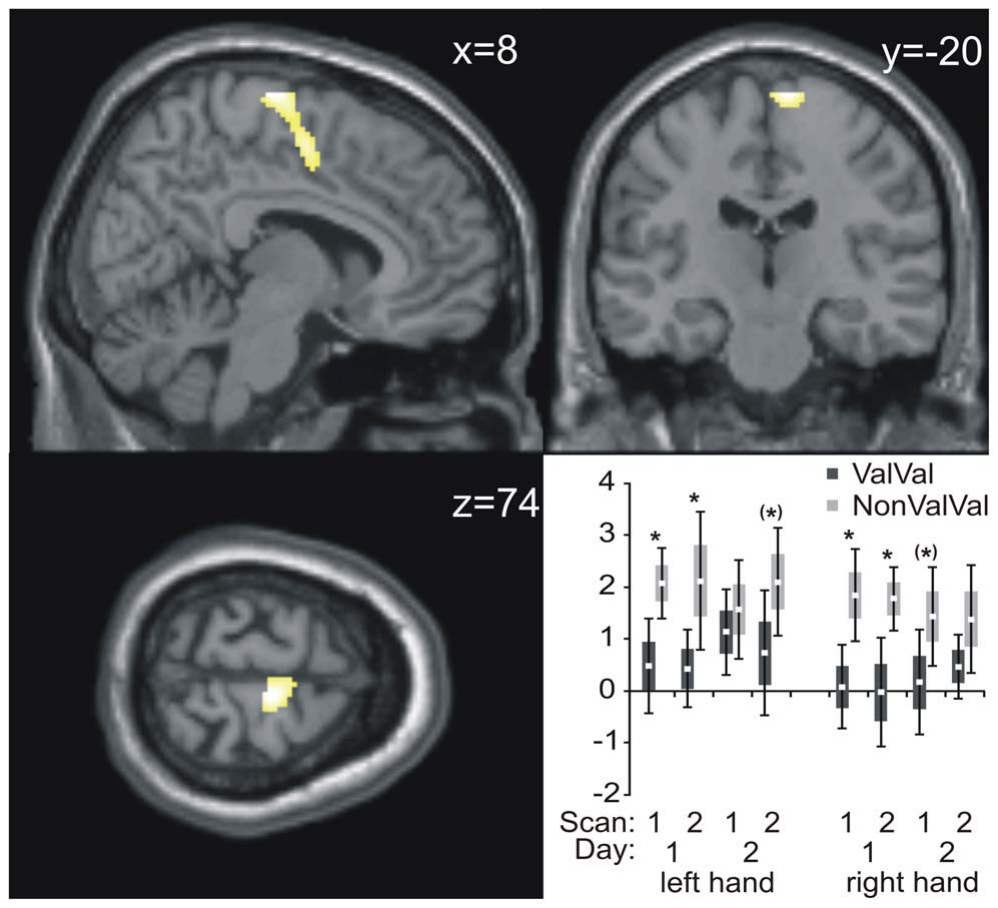
Significant group differences in BOLD signal for the contrast NonValVal > ValVal (t-test). Coordinates of the three sections depict the position of the peak voxel. In the inset, bar charts from mean activity of the peak voxel, separately for both hands are depicted for all 4 scans over two days (white squares: mean, grey bars: SEM, whiskers: 1.96*SEM). Ordinate: parameter estimates of neural magnitudes;*: p<0.0281, (*): p<0.05.

Appropriate t-tests on group differences for both hands and all sessions revealed significant (p<0.001; extent threshold of continuously significant voxels was set to 430 to obtain clusters significant at p<0.05) effects within the supplementary motor area (SMA) adjoining the middle cingulate gyrus (peak voxel x = 8, y = −20, z = 74, Z-score 3.9, Figure 1). Inspection of parameter estimates showed that this effect was due to the Non-ValVal group's higher BOLD-signal activity as compared to the ValVal group (the inverted t-contrast did not yield any significant effects). Additionally, the group's mean neural activity (parameter estimates) bearing significant group differences are depicted as bar charts in the subplot of Figure 1. Stability of group differences over time was explicitly tested using *post-hoc* two-sample *t*-tests. For these tests the nominal level of significance of *p*<0.05 was adjusted for multiple comparisons by means of a rough false discovery correction [19] according to the 8 (2 sessions x 2 scans x 2 hands) different two sample *t*-tests. Significance was inferred in the presence of p-values below the adjusted level of *p* = 0.0281. The group differences were consistent across fMRI sessions on day one, and kept the pattern of difference on day two although not all p values survived strict statistical control for multiple comparisons.

#### Correlation

No correlation was found in any session between behavioral data, the reaction times, and BOLD activity in the SMA peak voxel. Similarly, the parameters of cortical excitability, RMT and AMT did not correlate with reaction times.

## Discussion

In the present study we investigated two groups of healthy male subjects differing in the Val66Met SNP in the human BDNF gene. Neural activity upon left and right hand motor performance during a rather simple two-alternative forced choice task was measured with fMRI. Compared to ValVal subjects, Non-ValVal carriers showed significantly higher neural activity in SMA, adjoining the middle cingulate gyrus. The group differences were consistent across fMRI sessions performed at two days. In contrast, no behavioural differences were observed across groups, neither in terms of reaction times obtained from the fMRI task, nor in terms of indicators of cortical excitability obtained from electrophysiological data measured by single pulse TMS. Due to this discrepancy between activation and behavioural data this finding suggests the presence of cumulative effects of the polymorphism on the motor system that may reflect compensatory functional activation mediating equal behavioral performance between BDNF genotype groups.

### Electrophysiological data at rest (MT, RT)

Absence of group differences in cortical excitability measurements at rest using single pulse TMS (RMT and AMT) is in line with previous studies [10], [11]. Since the Val66Met polymorphism affects activity-dependent BDNF rather than constitutive BDNF secretion, this result was already to expect a priori, supporting the assumption that variations in the "met" polymorphism are activity dependent. TMS studies demonstrated reduced cortical excitability of M1 in Non-ValVal carriers when a longer interval of a 30 min finger training was part of the experimental protocol [11], as well as a lack of response to excitatory and inhibitory rTMS [10], [20]. From these studies it was suggested that Non-ValVal carriers may have higher thresholds to undergo synaptic plasticity processes and are therefore less susceptible to such changes than ValVal subjects. In contrast, a recent TMS study showed no differences between BDNF polymorphisms after excitatory and inhibitory theta-burst rTMS protocols [21]. It must be considered that other inter- and intra-cellular factors (e.g. glutamate and GABA release, growth factors such as IGF-1 or VEGF and Ca+2 binding proteins) might affect cortical excitability and motor plasticity (for reviews see [10], [22]). An increased intracortical excitability assessed by paired-pulse TMS has been observed in carriers of SNPs of the NMDA receptor and the glutamate regulator channel called transient receptor potential vanilloid 1 [23], [24].

### Simple choice motor reaction time task performance

Reaction times on button presses forced by our two-alternative task did not differ between groups, and absence of group differences was again stable over time. This aligns with previous studies that reported a lack of a BDNF polymorphism effect on peak velocity after short-term finger motor learning [11], [25]. Accordingly, it has been suggested that BDNF gene variation might selectively modulate the more cognitive aspects of any reaction time task (e.g. error related processing important in situations requiring behavioral adaptation) but not the general motor response per se [26]. However, again total practice time may also play a role in observing effects of the BDNF polymorphism on motor performance. For example, Fritsch et al. [3] compared performance of ValVal and Non-ValVal carriers after five days of motor learning. All participants started with similar baseline performance during the first learning session, but carriers of at least one Met allele displayed reduced motor skill acquisition as compared to ValVal carriers at the end of the last session. A second study used a driving-based motor task for 15 min, and observed fewer mistakes and better retention after four days of training in ValVal carriers as compared to the Non-ValVal group [12].

### fMRI BOLD-signal and BDNF polymorphisms during motor activity

In contrast to the reaction time data we observed a polymorphism effect on neural activity in the right SMA adjoining the middle cingulate gyrus. This effect appeared reliably over time and was present for involvement of either hand. SMA activity has consistently been observed during self-paced finger movement and simple choice reaction time tasks [27]–[31].

Only very little is known about differences in neural activation of BDNF genotypes during motor activity. McHughen et al. [12] suggested that the Val66Met polymorphism alters short-term plasticity in healthy volunteers (n = 24). Analysis across all subjects found a significant volume reduction within primary sensorimotor cortex to SMA across a period of 25 min of right index finger abduction/adduction training. However, ValVal subjects showed sites of greater activation expansion at bilateral sensorimotor cortex as compared to Non-ValVals at baseline and after training. In a separate experiment within the same study, subjects performed a driving-based motor task for 15 min. No changes in BOLD-signal between ValVal and Non-ValVal carriers were reported and all participants learned similarly; however, ValVal carriers committed fewer mistakes [12].

In our fMRI data differences in BOLD-signal across all scans might indicate that Non-ValVal carriers (have to) invest more neural effort to perform successfully a specific task as compared to ValVal carriers. We did not find significant correlations between performance (evaluated with reaction times) and BOLD-signal. Thus, our data suggest that Non-ValVal carriers show increased use of remote areas involved in motor function as a compensatory mechanism. In line with this finding, reduced hippocampal activation and higher caudate nucleus activity was observed in carriers of the Met BDNF polymorphism during fMRI of a human virtual navigation task [13]. This suggests that the availability of strategies based on different memory systems models the strategies available in people's everyday lives, and demonstrates compensatory mechanisms used by Met carriers. The underlying mechanisms of this possible compensation have not been explored. Recently, we demonstrated a negative correlation between the increase in MEP amplitudes after excitatory M1-rTMS and the baseline BOLD-signal in M1 in healthy volunteers [32]. Moreover, those subjects who showed a better response to rTMS featured stronger pre-interventional effective connectivity between SMA and ventral premotor cortex and left M1. Thus, it could be that the differences in BOLD-signal between ValVal and Non ValVal carriers in the present study are based on different patterns of neural connectivity. Furthermore, some of the genotype-related differences observed in fMRI data could reflect polymorphism effects on BDNF-related modulation of cortical inhibitory and excitatory processes [33]. For example, BDNF has been shown to facilitate glutamate release at a pre- and post-synaptic level [34]. There is also evidence for an effect of the BDNF Val66Met polymorphism on the glutamate system in the human hippocampus obtained from fMRI spectroscopy [35]. Carriers of the Met allele exhibited lower N-acetyl aspartate/creatine and glutamate/glutamine metabolic ratios. Thus, it could be that the decreased activity-dependent BDNF in the Non-ValVal carriers impacts on glutamatergic activity resulting in increased effort to perform simple motor tasks and therefore higher BOLD-signaling in combination with an extended use of more remote brain regions to perform an even simple motor task.

In summary, the present data support the concept of higher neural activation in carriers of the BDNF “Met” polymorphism within the motor system. The activation differences relative to Val/Val carriers were reliable over time and may reflect compensatory functional activation mediating equal motor performance, although the latter is still to be tested, for example, by virtual lesions induced by TMS of the SMA. Another limitation of the present study is small sample size of 11 subjects in each of the two genetically defined groups, and we fully acknowledge that actual effects await further replication at much larger sample sizes. We also did not control for the presence of other SNPs that could be involved in motor preparation and interact with performance. Several processes might influence execution of simple motor tasks, e.g. dopamine genetics as previously reported [36]. Further studies analyzing gene x gene interactions and effects on neuroplasticity would be helpful to accurately elucidate activation differences between subjects.
